# Candidate Gene Approach for Parasite Resistance in Sheep – Variation in Immune Pathway Genes and Association with Fecal Egg Count

**DOI:** 10.1371/journal.pone.0088337

**Published:** 2014-02-12

**Authors:** Kathiravan Periasamy, Rudolf Pichler, Mario Poli, Silvina Cristel, Bibiana Cetrá, Daniel Medus, Muladno Basar, Thiruvenkadan A. K., Saravanan Ramasamy, Masroor Babbar Ellahi, Faruque Mohammed, Atanaska Teneva, Mohammed Shamsuddin, Mario Garcia Podesta, Adama Diallo

**Affiliations:** 1 Animal Production and Health Laboratory, Joint FAO/IAEA Division of Nuclear Techniques in Food and Agriculture, International Atomic Energy Agency, Vienna, Austria; 2 Instituto de Genética “Ewald A. Favret”, Instituto Nacional de Tecnología Agropecuaria, Buenos Aires, Argentina; 3 Anguil Experimental Station, Instituto Nacional de Tecnología Agropecuaria Santa Rosa, La Pampa, Argentina; 4 Mercedes Experimental Station, Instituto Nacional de Tecnología Agropecuaria Mercedes, Corrientes, Argentina; 5 Concepción del Uruguay Experimental Station, Instituto Nacional de Tecnología Agropecuaria Concepción del Uruguay, Entre Ríos, Argentina; 6 Department of Animal Sciences, Bogor Agricultural University, Bogor, Indonesia; 7 Veterinary College and Research Institute-Namakkal, Tamil Nadu Veterinary and Animal Sciences University, Chennai, India; 8 Department of Animal Genetics and Breeding, University of Veterinary and Animal Sciences, Lahore, Pakistan; 9 Department of Animal Breeding and Genetics, Bangladesh Agricultural University, Mymensingh, Bangladesh; 10 University of Forestry, Sofia, Bulgaria; Instituto de Higiene e Medicina Tropical, Portugal

## Abstract

Sheep chromosome 3 (Oar3) has the largest number of QTLs reported to be significantly associated with resistance to gastro-intestinal nematodes. This study aimed to identify single nucleotide polymorphisms (SNPs) within candidate genes located in sheep chromosome 3 as well as genes involved in major immune pathways. A total of 41 SNPs were identified across 38 candidate genes in a panel of unrelated sheep and genotyped in 713 animals belonging to 22 breeds across Asia, Europe and South America. The variations and evolution of immune pathway genes were assessed in sheep populations across these macro-environmental regions that significantly differ in the diversity and load of pathogens. The mean minor allele frequency (MAF) did not vary between Asian and European sheep reflecting the absence of ascertainment bias. Phylogenetic analysis revealed two major clusters with most of South Asian, South East Asian and South West Asian breeds clustering together while European and South American sheep breeds clustered together distinctly. Analysis of molecular variance revealed strong phylogeographic structure at loci located in immune pathway genes, unlike microsatellite and genome wide SNP markers. To understand the influence of natural selection processes, SNP loci located in chromosome 3 were utilized to reconstruct haplotypes, the diversity of which showed significant deviations from selective neutrality. Reduced Median network of reconstructed haplotypes showed balancing selection in force at these loci. Preliminary association of SNP genotypes with phenotypes recorded 42 days post challenge revealed significant differences (P<0.05) in fecal egg count, body weight change and packed cell volume at two, four and six SNP loci respectively. In conclusion, the present study reports strong phylogeographic structure and balancing selection operating at SNP loci located within immune pathway genes. Further, SNP loci identified in the study were found to have potential for future large scale association studies in naturally exposed sheep populations.

## Introduction

Assessment of livestock health conditions in developing countries for identification of priority diseases to be targeted for control, revealed helminth infections as one of the most important problems in sheep and goat [1], [2]. Gastro-intestinal parasitic infestations such as *Haemonchus contortus, Teledorsagia circumcincta, Trichostrongyles, Nematodirus sp.* impose severe constraints on sheep and goat production especially those reared by marginal farmers under low external input system. These parasites incur heavy losses to farmers in terms of body weight loss, direct cost of anthelminthic drugs, loss due to mortality, etc. For example, annual treatment cost for *Haemonchus contortus* alone had been estimated to be 26 million USD in Kenya, 46 million USD in South Africa and 103 million USD in India [3]. Emergence of strains resistant to anthelminthic drugs has further complicated the management of parasitic diseases in small ruminants [4], [5]. Breeding programs with the goal of enhancing host resistance to parasites may help to alleviate this problem in the long term. Genetic variation in host resistance exists for the major nematode species affecting sheep: *Haemonchus contortus*, *Trichostrongylus colubriformis*, *Teledorsagia circumcincta* and various *Nematodirus* species. Considerable variation has been reported among sheep breeds on their ability to resist gastro-intestinal nematodes (GIN). For example, indigenous sheep breeds like Red Maasai [6], Garole [7], Gulf Coast Native [8], Rhon [9] and Barbados Black Belly [10] were found to have relatively better resistance against GINs. Similarly, within-breed genetic variation has also been demonstrated in diverse sheep populations including Merino [11], Romney [12], Scottish Blackface [13], feral Soay sheep [14], etc. Estimation of genetic parameters revealed low to moderate heritability in different sheep populations (h^2^ = 0.149, Avikalin sheep [15] to h^2^ = 0.41, Armidale sheep [16]).

Exploration of genetic variation either within specific regions of genome or more specifically in candidate genes involved in innate and adaptive immune pathways may help to identify a set of DNA markers significantly associated with parasite resistance characteristics. The former approach in terms of quantitative trait locus (QTL) analysis is a powerful method to understand genotype-phenotype relationship. Several QTL studies on parasite resistance characteristics have been reported in sheep. A quick evaluation of Animal QTL database [17] revealed a total of 753 QTLs reported for various economic traits in sheep. Among these, 81 were found to be related to parasite resistance characteristics and distributed in all sheep chromosomes except chromosomes 5 and 19. However, such QTLs related to parasite resistance were found to be more concentrated in chromosome 3 (16 QTLs) followed by chromosome 14 (7 QTLs). Among different parasites, 44 of 81 QTLs have been reported on resistance to Haemonchus spp., 20 on Trichostrongyles spp., 11 on Nematodirus spp. and six on Strongyles spp (Figure S1a–e). Thus the complexity of this analysis is evident from the fact that multiple, significant QTL regions have been reported across the entire genome, but the identification of candidate causative genes has remained elusive. The lack of consensus overlap among reported QTLs has hindered the identification of candidate genes and genetic markers for selection in sheep [18]–[21].

One of the important objectives of QTL studies is to identify underlying causative gene polymorphisms associated with the trait. Different QTLs reported in chromosome 3 for parasite resistance characteristics were found to be distributed all over the chromosome with varying overlapping regions. Hence, different candidate genes within chromosome 3 along with genes involved in immune related KEGG pathways (KEGG-Kyoto Encyclopedia of Genes and Genomes) could be important targets for establishing underlying causative variations. It is expected that the potential causative polymorphisms within candidate genes are members of the same overarching KEGG pathway that lead to the phenotypic expression on parasite resistance characteristics in each population. Further, the extent of genetic diversity and population sub-structure at such polymorphic loci are critical for such a genotype-phenotype association study. Population stratification has been demonstrated to result in false positive associations in various species including humans [22], [23], dogs [24] and cattle [25], [26]. Considering the significance of genetic basis of parasite resistance in sheep, the Joint FAO/IAEA Division of International Atomic Energy Agency initiated a coordinated research project to document the phenotypic differences and underlying genetic variations in indigenous sheep and goat breeds of 12 countries from Asia, Africa and South America through on-farm artificial challenge and natural exposure under farmers' field conditions respectively. The ultimate goal of this project is to identify a common set of genetic markers that significantly influence parasite characteristics across different indigenous populations of sheep and goats. The present study thus aimed at exploring genetic variations within genomic regions containing significant QTLs and different candidate genes involved in immune pathways, identification of single nucleotide polymorphisms (SNPs) and genetic diversity analysis in sheep populations evolved under different environmental conditions. A preliminary study was performed on association of genotypes with host resistance characteristics against gastro-intestinal nematodes (i.e. fecal egg count, body weight change and packed cell volume measured in response to artificial challenge with infective L3 larvae of *Haemonchus contortus* parasite).

## Materials and Methods

### Animal Ethics Statement

All procedures for artificial challenge experiment at different locations in Argentina and Indonesia were approved respectively by the Institutional Committee for Care and Use of Experimental Animals of the National Institute of Agricultural Technology (CICUAE-INTA), Argentina (protocol number 35/2010) and Institutional Research Animal Facility, Bogor Agricultural University, Indonesia following the guidelines described in their institutional manuals. Experimental animals were challenged with infective L3 larvae of *Haemonchus contortus* and blood samples were collected from jugular vein under the supervision of qualified veterinarians for extraction of DNA and assessment of blood parameters. 42 days post challenge, animals were dewormed to clear parasites from the gut after the experiment. The experimental challenge in both locations did not involve animals from any endangered or protected species/breeds. Blood sample collection for DNA extraction and genotyping from remaining breeds were performed by local veterinarians in respective countries following good animal practice.

### DNA samples for diversity analysis and association studies

**Figure 1:**
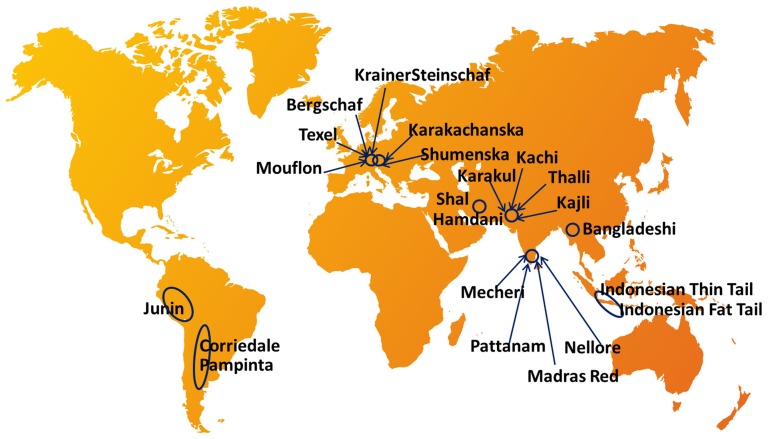
Location of sheep breeds sampled for the present study.

A total of 713 unrelated sheep from 22 different breeds/populations were utilized for diversity analysis in the present study. The sheep breeds/populations were distributed in three macro-environmental geographical locations including Asia (tropics), Europe (temperate) and South America (tropics) with the assumption that level of parasite load and infections vary significantly across these regions [27], [28]. The number of samples included for analysis from different breeds were as follows: Corriedale (102), Pampinta (34), Krainersteinschaf (42), Texel (21), Bergschaf (17), Mouflon (5), Karakachanska (20), Shumenska (14), Bangladeshi (17), Madras Red (60), Mecheri (64), Pattanam (54), Nellore (52), Indonesian Fat Tail (17), Indonesian Thin Tail (19), Shal (22), Hamdani (46), Thalli (17), Kachi (15), Karakul (17), Kajli (13) and Junin (45). The location of each of the sheep breeds/populations under study are presented in Figure 1. Artificial challenge experiments were carried out in two sheep breeds each from Argentina (Pampinta and Corriedale) and Indonesia (Indonesian Fat Tailed and Indonesian Thin Tailed) to generate phenotypes and DNA samples from these experimental animals were utilized for association study. Under artificial challenge trial, animals were dewormed before challenging with infective L3 larvae. Four to six weeks after deworming, blood samples were collected for DNA extraction and estimation of anemic parameters and experimental animals were challenged with a dose of 5000 infective L3 larvae cultured under *in vitro* conditions. All animals were maintained together during the entire trial in dry lot or under conditions of minimum additional parasite challenge. Body weight (BW), fecal egg count (FEC) and packed cell volume (PCV) were recorded at 0, 28, 35 and 42 days after artificial infection. 136 animals from Corriedale (66), Pampinta (34), Indonesian Fat Tail (17) and Indonesian Thin Tail breeds of sheep (19) were utilized for artificial challenge at experimental stations located in Argentina and Indonesia respectively.

### Targeted re-sequencing, SNP identification and Genotyping

A total of 39 candidate genes were identified for targeted re-sequencing and to detect SNPs. The candidate genes were selected based on analysis of global list of sheep Entrez Gene IDs in bovine KEGG database using KEGGARRAY to identify candidate genes involved in pathways related to immune system. Oligonucleotide primers were designed for PCR amplification of partial regions of genes in a panel of eight or 16 unrelated animals from different breeds located in major geographical regions under study. Sequences generated from both ends were edited using Codon Code Aligner version 3.7.1 and secondary peaks were called to ascertain SNPs. The forward and reverse sequences were assembled to generate contigs using BioEdit version 7.1.3 (http://www.mbio. ncsu.edu/bioedit/bioedit.html). 44 novel SNPs were identified within the candidate genes under study for which competitive allele specific PCR (KASPar) assay based on FRET chemistry were developed for genotyping (KBiosciences, LGC Genomics, UK). Briefly, two forward primers one specific to each allele were designed with the respective proprietary tail sequence complementing the FAM or HEX fluorescence reporting system. A common reverse primer was designed for each genotyping assay. Thermal cycling parameters and recycling conditions were followed as per manufacturer's recommendations and are available on request. Endpoint allele discrimination module incorporated within the BioRad CFX96 (BioRad, USA) was utilized for calling the genotypes based on fluorescent intensity recorded for each of the two alleles. The emission data of all the samples in the plate were plotted in X and Y axis respectively for each allele and the genotypes were called based on distinct clustering. Quality of allele calling was confirmed by comparing the genotypes derived from KASPar assay with the available sequence data on individuals from the panel of unrelated animals. 38 out of 44 assays passed quality control and were subsequently utilized for genotyping large number of animals. Additionally, ten toll like receptor (TLR) genes were selected for *in silico* mining of SNP variations from sequences available at NCBI-GenBank database. A total of 14 non-synonymous SNPs within coding DNA regions of TLR genes were identified for development of genotyping assays (Details of SNPs identified and reference sequences used from NCBI-GenBank are given in Table S1). However, only three of these SNPs (within TLR5, TLR7 and TLR8 genes) were found to be polymorphic, while the remaining 11 were monomorphic in the populations under present study. A total of 41 SNPs were finally utilized for diversity analysis and association with parasite resistance characteristics.

### Statistical Analysis

Basic diversity indices like allele frequency, genotype frequency, expected heterozygosity and test for Hardy Weinberg equilibrium were calculated using PEAS (Package for Elementary Analysis of SNP data) [29]. Allele sharing genetic distances (based on identical by state (IBS)) among pairs of individuals within and across different sheep breeds/populations were estimated using PEAS. Pair-wise allele sharing distance across different populations was utilized to construct the radial tree following UPGMA algorithm using PHYLIP version 3.5 [30]. Global F-statistics and pair-wise F_ST_ among different sheep breeds were computed using FSTAT software [31]. To investigate the sub-population structure of sheep breeds, pair-wise F_ST_ values among different sheep breeds were utilized to perform principal component analysis (PCA) using SPSS version 13.0. The first three principal components were used to draw the scattergram so as to understand underlying genetic structure and relationship of different breeds in three dimensional geometric space. The extent of population sub-structure was further explored using STRUCTURE with the assumption of different clusters, K = 1–15, 20, 25 and 30. Five replicate runs were performed for each K under admixture model without *a priori* population information. The number of burn in periods and MCMC repeats used for all the runs were 50000 and 100000 respectively. To identify the optimal ‘K’, the second order rate of change of L(K) with respect to ‘K’ was calculated by following the procedure reported elsewhere [32]. The results of STRUCTURE analysis were visualized using DISTRUCT [33].

Thirteen SNP loci within candidate genes involved in different immune related KEGG pathways and located in chromosome 3 were used to reconstruct haplotypes from unphased genotypic data. Reconstruction of haplotypes and estimation of haplotype frequencies were performed using PHASE for Windows, version 2.1 (www.stat.washington.edu/stephens) [34], [35]. The haplotype diversity and tests for departure from neutrality, Tajima's D, Fu and Li's D and Fu and Li's F were computed using DnaSP, version 4.10 [36]. The phased haplotypes were also utilized to perform analysis of molecular variance (AMOVA) and generate pair-wise F_ST_ values using ARLEQUIN version 3.1 [37]. Reduced Median network of haplotypes was constructed using NETWORK 4.5.1.2 with reduction threshold (r) of 10.0 [38].

A complete fixed effect model was employed for association of different genotypes at each SNP locus with fecal egg count (FEC), body weight change (BWC) and packed cell volume change (PCVC) measured 42 days post infective L3 larvae challenge. The data on FEC were subjected to log transformation before applying the program of least squares, LSMLMW [39]. The model included location of experimental stations and genotypes as fixed effects along with linear regression of breed effect on fecal egg count.

## Results and Discussion

### Immune pathway genes and SNP Discovery

A total of 243 sequences were generated by targeted re-sequencing of selected candidate genes (accession numbers are presented in Table 1) to identify 41 novel SNPs. Various details of SNPs including SNP ID, candidate gene name, chromosome location, genomic location, functional domain of the gene, alleles at each locus, SNP type, and strand genotyped are presented in Table 1. Among 41 SNPs identified, 27 were located in chromosome 3, the chromosome with maximum number of QTLs related to parasite resistance characteristics in sheep. Out of the remaining 14 SNPs, three were located in each of chromosomes 1 and 12, two in each of chromosomes 11, 16 and 27 and one in chromosome 8 and 13 respectively. The candidate genes selected for the study are involved in at least 18 KEGG pathways related to immune system (Table 2). The number of genes within each of these pathways varied from one to 14. JAK-STAT signaling pathway consisted a maximum of 14 genes followed by cytokine-cytokine receptor interaction and PIK3-AKT signaling pathways with eight genes each. The other major signaling pathways were Toll like receptor signaling pathway (5 genes) and chemokine signaling pathway (5 genes) followed by T cell receptor signaling pathway (4 genes). In a recent study, analysis of QTL and gene expression datasets following systems genetic approach revealed 14 KEGG pathways to be significant for parasite resistance in ruminants [21]. More than 50% of these immune related pathways have been included for candidate gene analysis in the present study. Altogether, 27 SNPs out of 41 SNP loci were within the candidate genes involved in immune pathways including 13 SNPs in chromosome 3. The location of SNPs in different functional domains of candidate genes varied considerably with 16 in 3′untranslated regions, 14 in exonic regions, 10 in intronic regions and one in 5′ flanking region upstream to start codon. Among the 14 SNPs located within exonic regions, eight were found to be non-synonymous mutations resulting in change of amino acid sequences while the remaining six were synonymous mutations.

**Table 1:** Details of candidate genes, chromosome location, genomic location and strand genotyped at 41 SNP loci under study.

SNPID	Accessions Nos.	Gene Name	Alleles	Chr	Position	Strand	Domain	Synon/Non-Synon
PIK3R3_498	KC734640-646	Phosphoinositide-3-kinase, regulatory subunit 3 (gamma)	T/G	1	20560912	+	Intron	-
LEPR_260	KC734602-617	Leptin receptor	G/A	1	42230500	+	Intron	-
IL6R_227	KC734807-813	Interleukin 6 receptor	T/C	1	110974347	−	3′UTR	-
ZDHHC17_190	KC734734-740	Zinc finger, DHHC-type containing 17	G/A	3	120038167	+	3′UTR	-
NAV3_591	KC734618-624	Neuron navigator 3	G/C	3	121343328	−	Exon	Synonymous
ACVRL1_445	KC734741	Activin A receptor type II-like 1	C/A	3	134236531	+	3′UTR	-
FGD6_519	KC734775-779	FYVE, RhoGEF and PH domain containing 6	G/A	3	140073272	−	Exon	G allele-D A allele-N
USP44_252	KC734721-727	Ubiquitin specific peptidase 44	T/C	3	140369758	+	Exon	C allele-G T allele-S
ITGA5_111	KC734594	Integrin, alpha 5 (fibronectin receptor, alpha polypeptide)	G/C	3	141110793	+	Intron	-
GPR84_520	KC734787	G-protein coupled receptor 84	G/A	3	141144005	+	5′ flanking region	-
TARBP2_97	KC734709-713	TAR (HIV-1) RNA binding protein 2	T/G	3	142063154	+	3′UTR	-
ITGB7_538	KC734595-601	Integrin, beta 7	G/A	3	142354366	+	Intron	-
CSRNP2_65	KC734757-761	Cysteine-serine-rich nuclear protein 2 (or) family with sequence similarity 130, member A1	T/C	3	144244455	−	Exon	Synonymous
SLC11A2_174	KC734676-682	Solute carrier family 11 (proton-coupled divalent metal ion transporters), member 2	T/C	3	144430523	+	3′UTR	-
PTPRB_141	KC734669-675	Protein tyrosine phosphatase, receptor type, B	G/A	3	158919598	+	Intron	-
STAT2_486	KC734684-688	Signal transducer and Activator of Transcription 2	G/C	3	162936891	+	3′UTR	-
DDIT3_527	KC734762-767	DNA-damage-inducible transcript 3	G/A	3	173067524	+	3′UTR	-
GLI1_253	KC734780-786	GLI family zinc finger 1	T/C	3	173101195	+	Exon	Synonymous
GLI1_576	KC734780-786	GLI family zinc finger 1	T/G	3	173101518	+	Exon	T allele-N G allele-H
ZBTB39_51	KC734728-733	Zinc finger and BTB domain containing 39	G/A	3	173591120	+	Exon	Synonymous
ANKRD52_113	KC734742-746	Ankyrin repeat domain 52	G/A	3	174303658	+	Intron	-
ESYT1_157	KC734769-774	Extended synaptotagmin-like protein 1 (or) family with sequence similarity 62 (C2 domain containing), member A	T/C	3	174409595	+	Intron	-
TIMP3_716	KC734714-720	TIMP metallopeptidase inhibitor 3	G/A	3	189483881	+	3′UTR	-
CSF2RB_279	KC734752-756	Colony stimulating factor 2 receptor, beta, low-affinity (granulocyte-macrophage)	T/G	3	193964280	+	Exon	Synonymous
CSF2RB_557	KC734752-756	Colony stimulating factor 2 receptor, beta, low-affinity (granulocyte-macrophage)	T/C	3	193964558	+	Exon	C allele-P T allele-L
IL2RB_180	KC734793-806	Interleukin 2 receptor, beta	T/C	3	194260690	-	Exon	Synonymous
EM4b_574	KC734768	EM4b protein	C/T	3	206971007	+	Intron	-
CLEC1A_134	KC734747-751	C-type lectin domain family 1, member A	T/C	3	220471614	+	Intron	-
PTPN6_398	KC734663-668	Protein tyrosine phosphatase, non-receptor type 6	G/A	3	224998785	+	Intron	-
SMCR7L_517	KC734683	Smith-Magenis syndrome chromosome region, candidate 7-like	G/A	3	233018827	−	3′UTR	-
IL20RA_422	KC734830-836	Interleukin 20 receptor alpha	G/A	8	66786499	−	Exon	G allele-G A allele-E
STAT5B_385	KC734694-708	Signal transducer and activator of transcription 5B	G/A	11	41843264	+	3′UTR	-
STAT3_138	KC734689-693	Signal transducer and activator of transcription 3 (acute-phase response factor)	G/A	11	44531311	+	3′UTR	-
IL10_82	KC734814-829	Interleukin 10	C/T	12	1856307	+	3′UTR	-
TLR5_2276	-	Toll like receptor 5	C/G	12	24704711	+	Exon	G allele-S C allele-T
PIK3CD_443	KC734625-639	Phosphoinositide-3-kinase, catalytic, delta polypeptide	G/A	12	46696545	−	3′UTR	-
IL2RA_388	KC734788-792	Interleukin 2 receptor, alpha	T/C	13	18432828	−	3′UTR	-
PRLR_341	KC734647-662	Prolactin receptor	C/T	16	42399902	+	3′UTR	-
PRLR_729	KC734647-662	Prolactin receptor	C/T	16	42400290	+	3′UTR	-
TLR7_2491	-	Toll like receptor 7	G/A	27	10360855	+	Exon	A allele-K G allele-R
TLR8_1045	-	Toll like receptor 8	T/C	27	10398449	+	Exon	T allele-Y C allele-H

**Table 2:** Details of candidate genes under study and involved in different KEGG immune pathways.

KEGG Path ID	KEGG Pathway	No. Genes	Candidate Genes
bta04630	Jak-STAT signaling pathway	14	CSF2RB,IL10,IL20RA,IL2RA,IL2RB,IL6R,LEPR,PIK3CD,PIK3R3,PRLR,PTPN6,STAT2,STAT3,STAT5B
bta04151	PI3K-Akt signaling pathway	8	IL2RA,IL2RB,IL6R,ITGA5,ITGB7,PIK3CD,PIK3R3,PRLR
bta04060	Cytokine-cytokine receptor interaction	8	CSF2RB,IL10,IL20RA,IL2RA,IL2RB,IL6R,LEPR, PRLR
bta04620	Toll-like receptor signaling pathway	5	PIK3CD,PIK3R3,TLR5,TLR7,TLR8
bta04062	Chemokine signaling pathway	5	PIK3CD,PIK3R3,STAT2,STAT3,STAT5B
bta04660	T cell receptor signaling pathway	4	IL10,PIK3CD,PIK3R3,PTPN6
bta04640	Hematopoietic cell lineage	3	IL2RA,IL6R,ITGA5
bta04650	Natural killer cell mediated cytotoxicity	3	PIK3CD,PIK3R3,PTPN6
bta04662	B cell receptor signaling pathway	3	PIK3CD,PIK3R3,PTPN6
bta04145	Phagosome	2	ITGA5
bta04520	Adherens junction	2	PTPN6,PTPRB
bta04664	Fc epsilon RI signaling pathway	2	PIK3CD,PIK3R3
bta04666	Fc gamma R-mediated phagocytosis	2	PIK3CD,PIK3R3
bta04670	Leukocyte transendothelial migration	2	PIK3CD,PIK3R3
bta04672	Intestinal immune network for IgA production	2	IL10,ITGB7
bta04010	MAPK signaling pathway	1	DDIT3
bta04142	Lysosome	1	SLC11A2
bta04340	Hedgehog signaling pathway	1	GLI1

### Minor allele frequency and genetic diversity within sheep breeds

The basic diversity measures estimated for different breeds under study: minor allele frequency, observed heterozygosity, expected heterozygosity and number of SNP loci deviating from Hardy-Weinberg equilibrium are presented in Table 3. The global minor allele frequency (MAF) across 41 SNP loci varied from 0.028 to 0.494 with a mean of 0.273. The power to detect genetic effect in a given study depends to a great extent on MAF of the tested alleles. Specifically, loci with a low MAF (<10%) have significantly lower power to detect weak genotype-phenotype associations than loci with a high MAF (>40%) [40], [41]. Further, previous studies have demonstrated that rare genotypes are more likely to result in spurious findings due to relatively higher standard error (within each test) and higher false discovery rate (under multiple testing procedures for many loci) [42]. In the present study, the global minor allele frequency was more than 0.10 in all but three SNP loci (FGD6_519, SMCR7L_517, TLR8_1045) thus indicating their suitability for association study. Examination of SNP loci within each breed revealed presence of both alleles in more than 90% of SNP loci, thus indicating high degree of polymorphism and possibility of these loci predating the radiation of sheep breeds under study. In addition, 49.8% of SNP loci showed MAF≥0.20 while 29.7% showed MAF≥0.30, suggesting the SNP set identified in the present study will likely have high utility for association analysis in different populations. The mean MAF within breeds varied from 0.167 (Pampinta) to 0.238 (Bangladeshi), although no significant difference in MAF was observed across different geographical regions: Asia, Europe and South America (Table 3). This is in contrast to earlier findings [43] which reported Asian and African breeds having excess of low MAF SNP (<0.10) compared to European populations. This reflects the absence of any ascertainment bias in the present study as the diversity panel was adequately represented with Asian sheep breeds for SNP discovery.

**Table 3:** Mean diversity indices and number of loci deviating from Hardy-Weinberg equilibrium in different sheep breeds at 41 SNP loci.

Population	Code	Indices of Genetic Diversity				
		MAF	Observed heterozygosity	Gene Diversity	No. Loci not in HWE	Mean IBS distance
**South Asian populations**						
Bangladeshi	BAN	0.238	0.282	0.314	8	0.272
Mecheri	MEC	0.194	0.266	0.262	6	0.206
Madras Red	MRS	0.202	0.295	0.282	4	0.229
Nellore	NEL	0.211	0.292	0.291	7	0.235
Pattanam	PAT	0.202	0.278	0.276	7	0.218
Kachi	KAC	0.204	0.234	0.279	12	0.253
Kajli	KAJ	0.193	0.278	0.262	10	0.221
Karakul	KUL	0.227	0.298	0.301	8	0.251
Thalli	THA	0.229	0.311	0.305	6	0.256
Mean		0.211	0.281	0.286	7.6	0.238
**South East Asian Populations**						
Indonesian Fat Tailed	IFT	0.209	0.262	0.282	4	0.250
Indonesian Thin Tailed	ITT	0.227	0.279	0.315	6	0.280
Mean		0.218	0.270	0.299	5.0	0.265
**South West Asian Populations**						
Hamdani	HAM	0.226	0.315	0.309	1	0.249
Shal	SHA	0.223	0.302	0.295	7	0.240
Mean		0.224	0.309	0.302	4.0	0.245
**European Populations**						
Krainersteinschaf	KSF	0.218	0.310	0.302	10	0.244
KarakaChanska	KAR	0.220	0.289	0.296	5	0.251
Shumenska	SHU	0.222	0.312	0.300	6	0.251
Bergschaf	BER	0.219	0.309	0.291	4	0.237
Texel	TEX	0.194	0.260	0.260	12	0.217
Mean		0.215	0.296	0.290	7.4	0.240
**South American Populations**						
Junin	JUN	0.218	0.299	0.304	4	0.250
Pampinta	PAM	0.167	0.230	0.237	7	0.197
Corriedale	COR	0.218	0.299	0.304	8	0.249
Mean		0.201	0.276	0.281	6.3	0.232

The mean global observed and expected heterozygosities were 0.287 and 0.366 respectively. The mean observed heterozygosity within breeds varied from 0.230 (Pampinta) to 0.315 (Hamdani) while the mean expected heterozyosity varied from 0.237 (Pampinta) to 0.315 (Indonesian Thin Tail). Among different geographical regions, mean observed heterozygosity was highest in South West Asian sheep populations (0.309) followed by European populations [0.296] while South East Asian populations had the least mean observed heterozygosity (0.270). This is consistent with the fact that the diversity remains higher around the centre of domestication while decreasing with increasing geographic distance [44]. Among the European sheep breeds, Texel, the northern Europe originated sheep breed was having the lowest mean observed heterozygosity (0.260) as compared to other South or South Eastern European breeds [45]. Further, the overall mean observed heterozygosity of South West Asian and European sheep populations was found to be higher than gene diversity, although similar case was observed with respect to most South Asian sheep populations except Bangladeshi, Kachi and Karakul. The test for HWE showed significant deviations with a mean number of loci 7.6, 5, 4, 7.4 and 6.3 in South Asian, South East Asian, South West Asian, European and South American sheep populations. Among all the sheep populations, Hamdani was found to be in equilibrium at all the SNP loci except one further reiterating its high degree of genetic diversity.

### Genetic distance within and between sheep breeds

**Figure 2:**
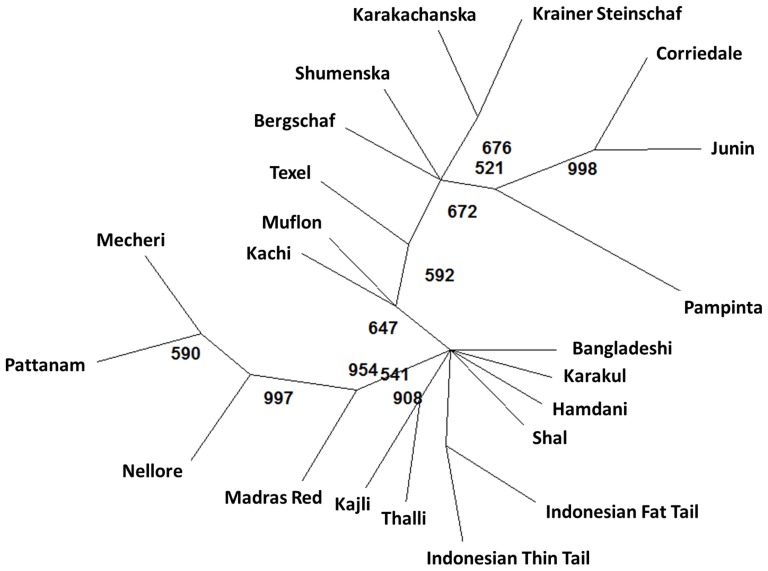
UPGMA radial tree constructed using pair-wise allele sharing distance among different sheep breeds. (Numbers at nodes indicate bootstrap values out of 10000 resampled datasets).

Allele sharing distance was calculated for all pair-wise combinations of individuals both within and across populations by subtracting average proportion of alleles shared from one [46]. The mean inter-individual allele sharing distance of all pair-wise combinations within breeds was 0.236 (SD = 0.06; n = 16907), while it ranged from 0.197 (Pampinta) to 0.280 (Indonesian Thin Tail). Across different geographical regions, the mean distance within breeds was lowest in South American populations (0.232) while it was highest in South East Asian populations (0.265). The mean distance between individuals derived from different breeds was 0.325 (SD = 0.07; n = 236921). Although the observed values were found to be higher than that reported for cattle, they were lower compared to the previous report in sheep [47]. The distribution of inter-individual distance values was found to be normal both within and across breeds (Figure S2). There was considerable overlap between inter-individual distances within and across breeds with almost equal proportion of pairwise combinations around the range of 0.28 to 0.30. This indicates that some individuals were found to be more closely related to individuals from other breed than from members of the same breed. To further investigate the genetic differentiation among different sheep breeds, pairwise allele sharing distance pair-wise F_ST_ (Table S2) and global F-statistics (Table S3) were estimated. The global F_IT_, F_ST_ and F_IS_ were 0.227, 0.213 and 0.018 respectively while pairwise F_ST_ values ranged from 0.017 (Pattanam/Nellore) to 0.469 (Pampinta/Mouflon). 21.3% of total genetic variation was found to be due to between breed differences while 77.3% was due to within breed differences. The values are much higher than that reported for European sheep (13.1%) [45], Indian sheep (11.1%) [48] and European and South West Asian sheep (5.7%) [44] using microsatellite markers. The higher F_ST_ values observed could be understood from the fact that the samples were derived from wide geographic locations in the present study (Asia, Europe and South America). Further, phylogenetic analysis of pair-wise allele sharing distance revealed two major clusters with most of South Asian, South East Asian and South West Asian breeds clustering together while the European and South American sheep breeds clustered together separately (Figure 2). However, the three South American sheep breeds formed a sub-cluster together and interestingly found to be more closely related to Southern Europe sheep breeds than North European sheep. Similarly Karakul and Bangladeshi breeds were found to be clustering together with South West Asian sheep than with other South Asian breeds.

### Genetic structure of sheep breeds

**Figure 3:**
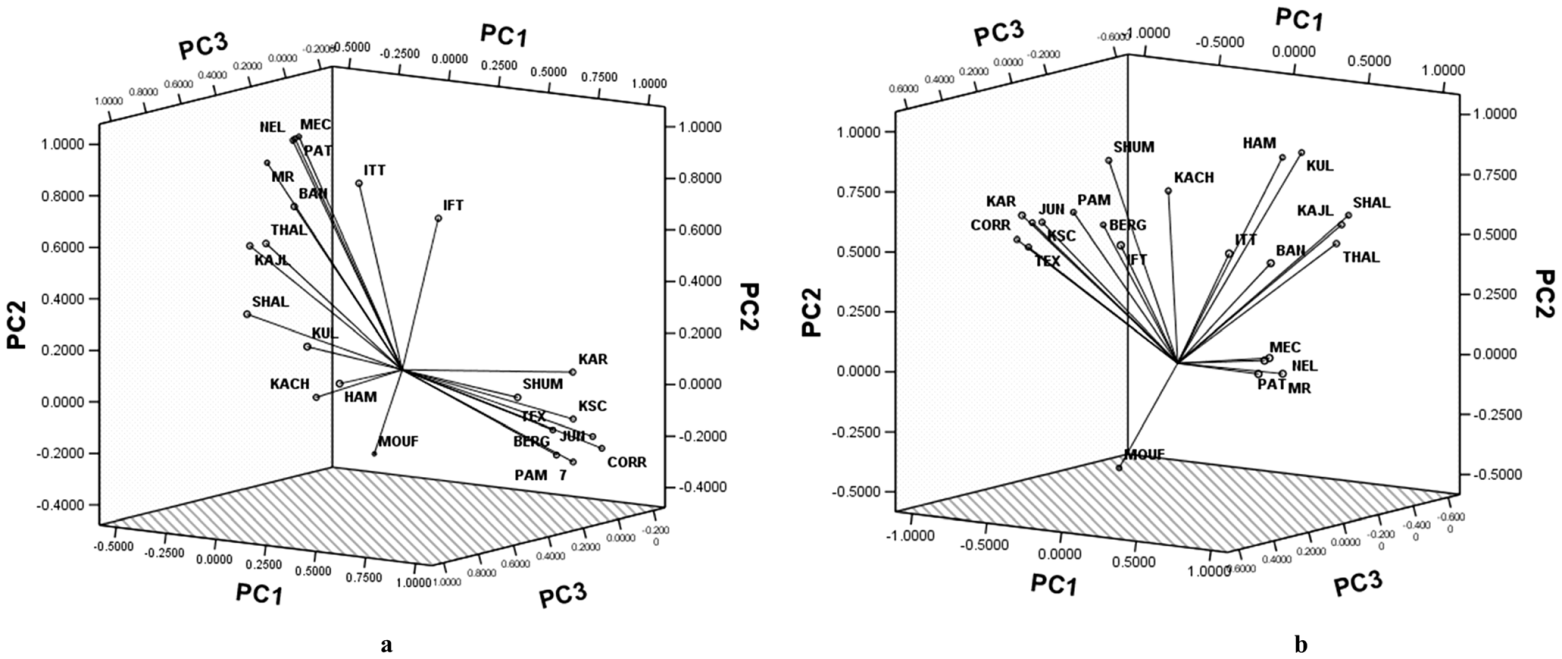
Three dimensional scattergram of first three principal components derived from pairwise F_ST_ (a) across 41 SNP loci and (b) across 27 SNP loci among different sheep populations. (BAN-Bangladeshi; COR-Corriedale; PAM-Pampinta; JUN-Junin; BER-Bergschaf; TEX-Texel; KSF-Krainersteinschaf; MUF-Mouflon; KAR-Karakachanska; SHU-Shumenska; KUL-Karakul; THA-Thalli; KAC-Kachi; KAJ-Kajli; HAM-Hamdani; SHA-Shal; PAT-Pattanam; NEL-Nellore; MRS-Madras Red; MEC-Mecheri; IFT-Indonesian Fat Tail; ITT-Indonesian Thin Tail).

**Figure 4:**
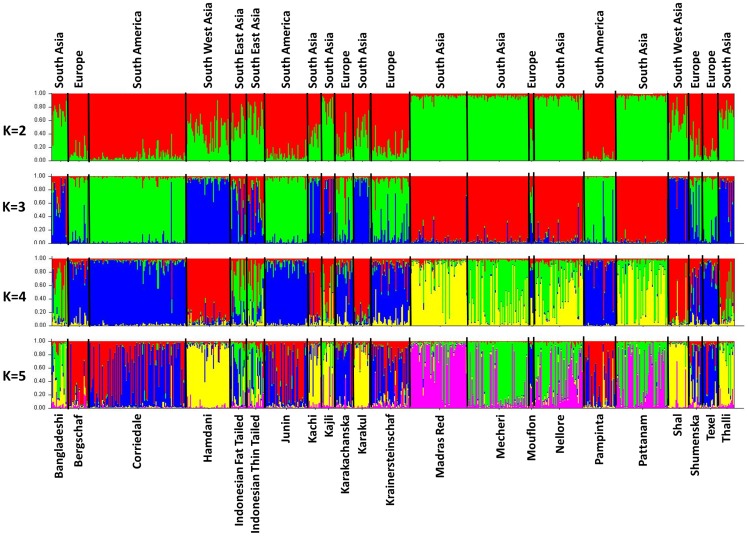
Bayesian clustering of 713 sheep under assumption of 2 to 6 clusters without a priori population information. The breed names are given below the box plot and the geographical origin indicated above the box plot with the individuals of different breeds separated by vertical black lines.

The pairwise F_ST_ were subjected to principal components analysis and the first three principal components (PCs) were plotted on a three dimensional scattergram to evaluate the genetic structure of sheep breeds (Figure 3a). The first, second and third principal components explained 42.01%, 38.12% and 7.15% of total genetic variation respectively. The clustering of sheep breeds followed their geographical origin and broadly differentiated into European and Asian groups. European Mouflon, which is more feral in nature was distinct from both these groups. However, analysis of a subset of SNP data at 27 loci located within immune pathway genes resulted in an additional distinct group with all the four South Indian sheep breeds clustering together closely (Figure 3b). In order to further understand the phylogeographic structure, analysis of molecular variance was performed to assess the SNP variation as a function of both breed membership and geographic origin (Table 4). Two types of groupings were assumed; the first grouping was with three major geographical groups Asia, Europe and South America; the second one with five geographical groups South Asia, South East Asia, South West Asia, Europe and South America. With grouping I, 14.16% of variation was due to differences in geographical groups and 11.54% due to between breed differences. In case of grouping II, among group variation marginally increased to 14.64% while between breed differences decreased to 9.51%. Further, with the analysis of subset of immune pathway SNP data at 27 loci, among group variation increased to 15.92% with grouping II showing a strong phylogeographic structure (Table 3). This is in contrast to earlier reports based on microsatellite genotypes [45], SNP genotypes [47] and mitochondrial DNA haplotypes [49], where much less variation was explained by grouping breeds into geographical regions. Similarly weak phylogeographic structure had been reported in domestic goats also [50]. All these studies concluded that the weak phylogeographic structure exhibited by domestic sheep and goat might be due to their small size and versatility enabling transportation and subsequent introgression in concert with human migration [47], [51]. The relatively strong phylogeographic structure observed in the present study is interesting from the fact that most of these SNP loci are within candidate genes involved in different immune pathways. The geographical locations of sampled individuals vary widely with respect to diversity and load of pathogens resulting in differences in the magnitude of natural selection pressure across these regions [27], [28]. Consequently, evolution of genes involved in immune system may either be highly optimized by natural selection process (purifying selection) or continue to evolve under low selection pressure (balancing selection) [52]. The genetic structure of sheep populations exhibited by a set of SNPs located in immune pathway genes could thus be different from those revealed by microsatellite or mitochondrial or genome wide SNP variations. In order to further clarify the breed demography and selection history, all the 41 SNP loci were subjected to Ewens-Watterson neutrality test to investigate whether the loci were influenced by selective forces within various sheep breeds/populations. 18 out of 41 SNP loci were found to deviate from selection neutrality in at least one of the breeds under study while the remaining 23 SNP loci were found to be selectively neutral (Table S4). The two subsets of data (23 neutral SNP loci and 18 non-neutral loci) were subjected to analysis of molecular variance. Among group variance at non-neutral SNP loci was found to be significant and higher (17.13%) than variance among populations within group as compared to selectively neutral SNP loci (11.01%) indicating the basis for strong phylogeographic structure observed in the present study (Table S5). Bayesian clustering was performed without prior population information using STRUCTURE program, and the second order rate of change of average likelihood at each K was calculated (K = 1–15, 20, 25, 30). ΔK reached its peak at K = 5, suggesting optimal K value appropriate for the dataset. When K = 2 was assumed, most of the individuals from Europe and South America were assigned to cluster 1, while all the four Indian breeds were assigned to cluster 2 (Figure 4). Individuals belonging to other breeds from South Asia (Kachi, Kajli, Karakul and Thalli), South West Asia (Hamdani, Shal) and South East Asia (Indonesian Fat Tail and Indonesian Thin Tail) were admixed and observed to be assigned in both the clusters. When K = 3 was assumed, European and South American sheep were mostly assigned to cluster 1, Indian sheep to cluster 2 and the South West Asian sheep along with Karakul to cluster 3. Indonesian sheep and other South Asian sheep were found to be admixed between cluster 2 and 3. With K = 4, the Indian sheep population got subdivided into two clusters while with K = 5, the subdivision of European cluster was evident. Further, Bayesian analysis was also performed with the subsets of genotypes at non-neutral and neutral SNP loci (Figure S3a and S3b respectively). The results revealed relatively better and more precise geographical clustering of animals with the non-neutral subset than the neutral subset of genotype data.

**Table 4:** Analysis of molecular variance among different sheep breeds based on (right) genotypes at 41SNP loci and (left) haplotypes reconstructed from genotypes at 13 immune SNP loci located in chromosome 3.

Source of variation	Based on genotype data at 41 SNP loci						Based on haplotypic data from 13 SNP loci					
	d.f.	Sum of squares	Variance components		% of variation	P- value	d.f.	Sum of squares	Variance components		% of variation	P-value
***No Groupings***												
Among populations	21	2205.51	1.57	Va	21.33	0.000	21	767.50	0.55	Va	22.22	0.000
Within populations	1404	8118.99	5.78	Vb	78.67	0.000	1404	2689.14	1.92	Vb	77.78	0.000
***Grouping-I (Asia, Europe, South America)*** *												
Among groups	2	1075.13	1.10	Va	14.16	0.000	2	349.20	0.34	Va	13.27	0.000
Among populations within groups	19	1130.38	0.90	Vb	11.54	0.000	19	418.30	0.34	Vb	12.94	0.000
Within populations	1404	8118.99	5.78	Vc	74.30	0.000	1404	2689.14	1.92	Vc	73.80	0.000
***Grouping-II (South Asia, South East Asia, South West Asian, Europe, South America)*** **												
Among groups	4	1370.78	1.16	Va	14.64	0.000	4	493.56	0.41	Va	15.92	0.000
Among populations within groups	17	834.73	0.72	Vb	9.51	0.000	17	273.94	0.24	Vb	9.27	0.000
Within populations	1404	8118.99	5.78	Vc	75.86	0.000	1404	2689.14	1.92	Vc	74.81	0.000

* Grouping-I: Asia – Bangladeshi, Madras Red, Mecheri, Pattanam, Nellore, Indonesian Fat Tailed, Indonesian Thin Tailed, Shal, Hamdani, Thalli, Kachi, Karakul, Kajli; Europe – Krainersteinschaf, Texel, Bergschaf, Mouflon, Karakachanska, Shumenska; South America – Junin, Pampinta, Corriedale.

** Grouping-II: South Asia - Bangladeshi, Madras Red, Mecheri, Pattanam, Nellore, Thalli, Kachi, Karakul, Kajli; South East Asia - Indonesian Fat Tailed, Indonesian Thin Tailed; South West Asia – Hamdani, Shal; Europe – Krainersteinschaf, Texel, Bergschaf, Mouflon, Karakachanska, Shumenska; South America – Junin, Pampinta, Corriedale.

### Haplotype reconstruction and test for neutrality

**Figure 5:**
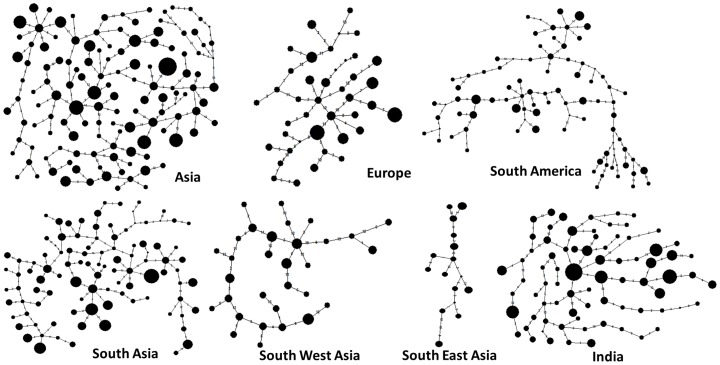
Reduced Median network of haplotypes reconstructed from 13 SNP loci related to immune genes in sheep chromosome 3. (Asia – Bangladeshi, Madras Red, Mecheri, Pattanam, Nellore, Indonesian Fat Tailed, Indonesian Thin Tailed, Shal, Hamdani, Thalli, Kachi, Karakul, Kajli; Europe – Krainersteinschaf, Texel, Bergschaf, Mouflon, Karakachanska, Shumenska; South America – Junin, Pampinta, Corriedale; South Asia - Bangladeshi, Madras Red, Mecheri, Pattanam, Nellore, Thalli, Kachi, Karakul, Kajli; South East Asia - Indonesian Fat Tailed, Indonesian Thin Tailed; South West Asia – Hamdani, Shal; India – Madras Red, Maecheri, Nellore, Pattanam).

To investigate the influence of natural selection processes, unphased diploid genotypes at SNP loci located in chromosome 3 were utilized to reconstruct the haplotypes. Out of 27 SNP loci located in chromosome 3, 13 loci within immune pathway genes were used for haplotype phasing. Data on all 713 animals were utilized to reconstruct a total of 1426 haplotypes, of which 389 were found to be singletons. Predicted haplotype phases with best pair probabilities for each individual were retained for further analysis. Table 5 provides the results of tests for selective neutrality using three different statistics: Tajima's D, Fu and Li's D and Fu and Li's F. Significant deviations were found in Corriedale, Junin, Krainer Steinschaf, Texel and Nellore sheep breeds. All the three statistics showed significant deviation from neutrality in Junin breed, while two statistics in each of corriedale and Texel sheep breeds and one statistics in each of Krainersteinschaf and Nellore sheep breeds showed significant deviations from selective neutrality. When tested at regional level, Tajima's D and Fu and Li's F statistics revealed significant deviations in European, South American and South Asian populations. Similarly, the Indian sheep which clustered distinctly when analyzed at immune SNP loci, showed significant deviation from neutrality under Tajima's D and Fu and Li's F tests. However, Fu and Li's D statistic did not detect any significant deviation from neutrality in all these populations. All the test statistics that showed significant deviation both at breed and regional levels were found to be positive indicating balancing selection in force, probably with low selection pressure. In order to further examine this process, haplotype networks were constructed for each of the geographical regions under study. Star contraction is expected under strong purifying selection while few haplotypes with moderate frequencies and short branches are expected under a weak purifying selection. On the contrary, balancing selection is expected to retain multiple lineages with high and low frequency clusters and long branches [53]. Reduced Median (RM) networks were constructed for haplotypes derived from populations in different geographical regions (Figure 5). All the populations showed multiple lineages with several nodes of different sizes and long branches, thus confirming balancing selection in force as revealed by different tests for neutrality. This is further evident from the fact that many breeds under study were found to have either heterozygosity excess or near equal observed and expected heterozygosities. Although little information is available in sheep on immune gene polymorphisms across distinct geographical regions, few reports are available in human and cattle. In a study on innate immune genes including TLRs and defensins in Indian, European-American and African-American human populations, strong purifying selection was found to operate resulting in conservation of recognition motifs across a broad range of pathogens [52]. Similar observation was found with respect to TLR10 gene in a study on *Bos taurus* and *Bos indicus* cattle [54]. However, in case of adaptive immune genes like major histocompatibility complex, balancing selection with high genetic diversity and heterozygote advantage was found to be common [55], [56]. To the best of our knowledge, the present study is the first to report balancing selection forces operating in immune pathway genes of sheep. However, it needs to be noted that there may be local variations in the nature of selection as it could be modulated by local differences in pathogen diversity and load [52].

**Table 5:** Values of haplotype diversity and statistics for testing departures from neutrality (Haplotypes reconstructed from 13 SNPs in chromosome 3-R Method).

Population/Breed	N	No. Haps	Haplotype diversity		Tajima's D		Fu and Li's D*		Fu and Li's F*	
			Estimate	Variance	Value	P	Value	P	Value	P
Bangladeshi	34	31	0.995	0.00007	1.82	P>0.05	0.92	P>0.10	1.41	P>0.10
Bergschaf	42	26	0.954	0.00043	0.87	P>0.10	1.02	P>0.10	1.15	P>0.10
Corriedale	204	131	0.992	0.00000	2.75	P<0.05	1.48	P>0.05	2.34	P<0.02
Hamdani	92	45	0.971	0.00004	0.50	P>0.10	0.93	P>0.10	0.92	P>0.10
Indonesian Fat Tailed	34	24	0.971	0.00022	0.73	P>0.10	0.92	P>0.10	1.01	P>0.10
Indoesian Thin Tailed	38	31	0.990	0.00007	1.30	P>0.10	0.97	P>0.10	1.27	P>0.10
Junin	90	68	0.991	0.00002	2.72	P<0.01	1.51	P<0.05	2.31	P<0.02
Kachi	30	18	0.940	0.00072	0.66	P>0.10	1.00	P>0.10	1.05	P>0.10
Kajli	26	22	0.985	0.00026	1.13	P>0.10	0.48	P>0.10	0.79	P>0.10
Karakachanska	40	35	0.991	0.00007	1.52	P>0.10	1.03	P>0.10	1.40	P>0.10
Karakul	34	25	0.979	0.00016	0.78	P>0.10	1.04	P>0.10	1.13	P>0.10
Krainer Steinschaf	84	53	0.972	0.00010	1.27	P>0.10	1.51	P<0.05	1.70	P>0.05
Madras Red	120	63	0.981	0.00001	1.56	P>0.10	0.81	P>0.10	1.29	P>0.10
Mecheri	128	49	0.964	0.00005	0.92	P>0.10	0.80	P>0.10	1.01	P>0.10
Mouflon	10	6	0.844	0.01060	0.17	P>0.10	0.64	P>0.10	0.59	P>0.10
Nellore	104	64	0.987	0.00001	1.95	P>0.05	1.42	P>0.05	1.91	P<0.05
Pampinta	68	48	0.986	0.00003	1.02	P>0.10	0.41	P>0.10	0.74	P>0.10
Pattanam	108	51	0.973	0.00003	1.49	P>0.10	0.75	P>0.10	1.20	P>0.10
Shal	44	24	0.967	0.00013	1.15	P>0.10	0.33	P>0.10	0.70	P>0.10
Shumenska	28	24	0.989	0.00015	1.08	P>0.10	1.01	P>0.10	1.21	P>0.10
Texel	34	26	0.964	0.00043	1.35	P>0.10	1.44	P<0.05	1.66	P<0.05
Thalli	34	29	0.988	0.00013	1.32	P>0.10	0.92	P>0.10	1.23	P>0.10
Europe	238	139	0.989	0.00000	2.66	P<0.05	1.47	P>0.05	2.31	P<0.02
South America	362	193	0.993	0.00000	3.10	P<0.01	1.44	P>0.05	2.51	P<0.02
South East Asia	72	51	0.987	0.00003	1.55	P>0.10	1.48	P>0.05	1.78	P<0.05
South Asia	628	194	0.987	0.00000	2.57	P<0.05	1.40	P>0.05	2.25	P<0.02
South West Asia	136	61	0.980	0.00001	1.13	P>0.10	0.87	P>0.10	1.15	P>0.10
India	470	142	0.982	0.00000	2.15	P<0.05	1.38	P>0.05	2.02	P<0.05

### Association of immune pathway gene polymorphisms with fecal egg count

**Figure 6:**
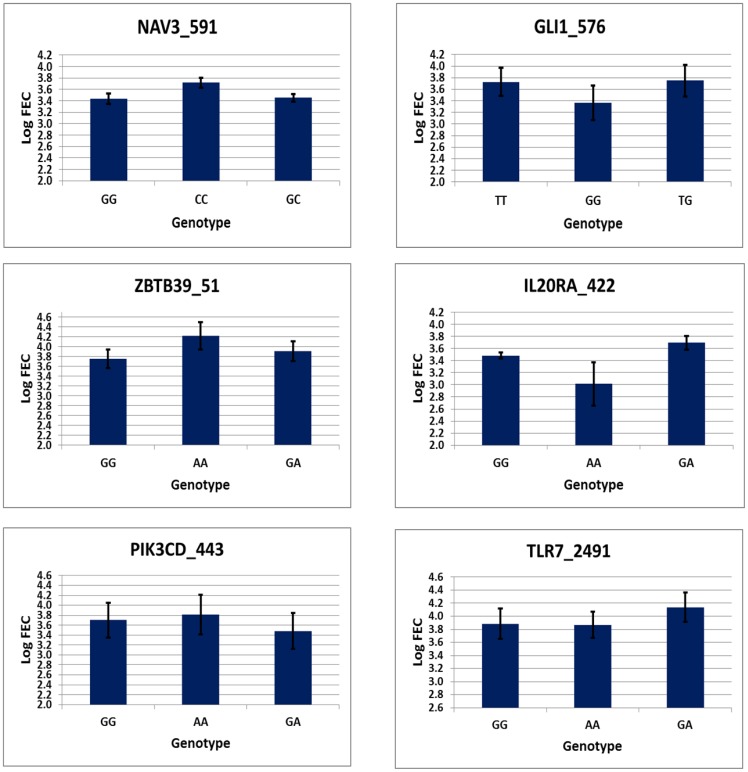
Least squares mean and standard error of log transformed fecal egg count 42 days post challenge at significant SNP loci in two Argentinian (Pampinta and Corriedale) and two Indonesian (Indonesian Thin Tailed and Indonesian fat tailed) sheep breeds.

To evaluate the potential utility of the SNP loci for future association study on a large number of samples, a pilot analysis was performed with the phenotypes generated in four breeds after artificial challenge with infective L3 larvae of *Haemonchus contortus*. Phenotypic data (fecal egg count, body weight change and change in packed cell volume 42 days post challenge) on 136 animals was used for least squares analysis under a complete fixed effect model. The effect of location of experimental stations (Corriedale and Pampinta in South America; Indonesian Thin Tail and Indonesian Fat Tailed sheep in South East Asia) was not found to have significant influence on fecal egg count and packed cell volume while significant effect was observed with respect to body weight change (P<0.01). Although the locations are wide apart geographically, uniform protocol was followed across different experimental stations in terms of age of lambs selected for experiment, deworming and data recording schedule, however some differences did exist in terms of quality of pasture available for grazing, etc. Higher observed body weight change in animals challenged at Aguil Experimental Station (AES), Argentina was due to better growth performance of Pampinta lambs. Higher body weight achieved by Pampinta lambs were due to their genetic differences in growth rate (average pre-weaning weight gain of 295 g/day) and weight gain (average weaning weight of 33.4 kg) as compared to other breeds like Corriedale (218 g/day and 24.6 kg) [57] and Indonesian Fat Tailed sheep (47.7 g/day and 9.7 kg) [58]. The fixed effect on body weight change was adjusted before phenotype-genotype association analysis. Among different breeds, lowest mean fecal egg count (mean log FEC 3.23±0.16) and packed cell volume change (−1.57%) was observed in Indonesian fat tailed sheep while Corriedale showed highest mean values for these traits (mean logFEC 3.58±0.079 and PCV of −4.97%), although the differences were not statistically significant (P>0.05). Among the SNP loci examined, genotypes at two loci, NAV3_591 and GLI1_576, both located in chromosome 3 and within exonic regions of the respective genes (Neuron navigator and GLI family zinc finger 1) were found to have significant differences in their fecal egg count (Table 6). Among these, GLI1_576 locus was a non-synonymous change from Asparagine (T allele) to Histidine (G allele). The mean log transformed fecal egg count at NAV3 locus was 3.435, 3.717 and 3.453 for GG, CC and GC genotype groups respectively. Similarly, the mean log transformed fecal egg count at GLI1_576 locus was 3.724, 3.364 and 3.749 for TT, GG and TG respectively (Figure 6). Apart from these two loci, genotypes at ZBTB39_51, IL20RA_422, PIK3CD_433 and TLR7_2491 showed weak differences in their mean log transformed fecal egg count, although statistically not significant (P<0.10). However, it needs to be mentioned that none of these loci were found to have significant association when multiple testing correction factors were applied using Benjamini-Hochberg false discovery rate (FDR corrected P- value>0.10 for all the SNP loci). Similarly, with respect to body weight change, four loci (ACVRL1_445, GPR84_520, TARBP2_97 and SMCR7L_517) located in chromosome 3 were found to have significant association (P<0.05) but with higher FDR values (P>0.05). Association of genotypes with packed cell volume change 42 days post challenge revealed significance at six SNP loci (NAV3_591, CSRNP2_65, ANKRD52_113, ESYT1_157, TIMP3_716 and IL2RA_388), of which ESYT1_157 showed significant FDR corrected p value of 0.029. The mean change in packed cell volume of genotypes TT, CC and TC at this locus were −7.96%, −6.09% and −4.02% respectively. With respect to SNP loci showing significant or weak association with phenotypes (including fecal egg count, body weight change and packed cell volume change), some showed heterozygous advantage while few others had no heterozygous advantage. Unlike haplotype analysis conducted on many breeds within each region that showed balancing selection and heterozygous advantage, association analysis was performed in few selected breeds. Considering the results from neutrality tests, selection influence at a particular SNP locus vary across breeds and hence the balancing selection observed in haplotype networks within a particular region might be due to different alleles being favoured within and across loci in various breeds. However, it has to be noted that the preliminary association in the present study is based on relatively fewer number of animals and expected heterozygous advantage has to be tested in large populations.

**Table 6:** Least squares ANOVA of parasite resistance characteristics (log transformed fecal egg count, body weight change and packed cell volume change 42 days post challenge at different SNP loci in experimentally challenged sheep (Pampinta and Corriedale, Indonesian Thin Tailed and Indonesian fat tailed).

SNPID	Chromosome	Fecal egg count		Body Weight		Packed cell volume	
		P-Value	FDR*	P-Value	FDR*	P-Value	FDR*
PIK3R3_498	1	0.385	0.727	0.584	0.940	0.797	0.846
LEPR_260	1	0.937	0.998	0.161	0.516	0.741	0.844
IL6R_227	1	0.776	0.998	0.716	0.955	0.377	0.736
ZDHHC17_190	3	0.167	0.727	0.222	0.607	0.296	0.736
NAV3_591	3	0.038	0.604	0.561	0.940	0.046	0.313
ACVRL1_445	3	0.209	0.727	0.018	0.331	0.716	0.839
FGD6_519	3	0.299	0.727	0.596	0.940	0.704	0.839
USP44_252	3	0.110	0.643	0.892	0.955	0.538	0.809
ITGA5_111	3	0.984	0.998	0.742	0.955	0.238	0.711
GPR84_520	3	0.317	0.727	0.024	0.331	0.062	0.317
TARBP2_97	3	0.506	0.830	0.045	0.463	0.055	0.317
ITGB7_538	3	0.890	0.998	0.795	0.955	0.315	0.736
CSRNP2_65	3	0.607	0.958	0.170	0.516	0.022	0.224
SLC11A2_174	3	0.314	0.727	0.924	0.955	0.805	0.846
PTPRB_141	3	0.998	0.998	0.923	0.955	0.260	0.711
STAT2_486	3	0.292	0.727	0.751	0.955	0.341	0.736
DDIT3_527	3	0.314	0.727	0.628	0.953	0.796	0.846
GLI1_253	3	0.344	0.727	0.267	0.684	0.105	0.432
GLI1_576	3	0.033	0.604	0.588	0.940	0.102	0.432
ZBTB39_51	3	0.062	0.604	0.066	0.516	0.637	0.839
ANKRD52_113	3	0.896	0.998	0.767	0.955	0.019	0.224
ESYT1_157	3	0.658	0.998	0.092	0.516	0.001	0.029
TIMP3_716	3	0.348	0.727	0.521	0.940	0.042	0.313
CSF2RB_279	3	0.414	0.739	0.436	0.862	0.695	0.839
CSF2RB_557	3	0.270	0.727	0.976	0.976	0.672	0.839
IL2RB_180	3	0.390	0.727	0.151	0.516	0.699	0.839
EM4b_574	3	0.138	0.707	0.403	0.862	0.451	0.803
CLEC1A_134	3	0.924	0.998	0.441	0.862	0.325	0.736
PTPN6_398	3	0.965	0.998	0.916	0.955	0.255	0.711
SMCR7L_517	3	0.739	0.998	0.001	0.057	0.992	0.992
IL20RA_422	8	0.085	0.604	0.932	0.955	0.410	0.764
STAT5B_385	11	0.916	0.998	0.176	0.516	0.360	0.736
STAT3_138	11	0.990	0.998	0.764	0.955	0.537	0.809
IL10_82	12	0.862	0.998	0.437	0.862	0.236	0.711
TLR5_2276	12	0.481	0.821	0.848	0.955	0.860	0.881
PIK3CD_443	12	0.088	0.604	0.100	0.516	0.248	0.711
IL2RA_388	13	0.823	0.998	0.166	0.516	0.006	0.113
PRLR_341	16	0.374	0.727	0.142	0.516	0.486	0.809
PRLR_729	16	0.955	0.998	0.822	0.955	0.509	0.809
TLR7_2491	27	0.069	0.604	0.335	0.808	0.572	0.809
TLR8_1045	27	0.313	0.727	0.106	0.516	0.572	0.809

* FDR – Benjamini-Hochberg False Discovery Rate corrected P-value.

In conclusion, the present study reports strong phylogeographic structure in sheep across Asia, Europe and South America and balancing selection operating at SNP loci located within immune pathway genes. Although the present association analysis is preliminary in nature, the SNP loci on chromosome 3 and those within immune pathway genes indicated their potential for future large scale association studies in naturally exposed populations.

## Supporting Information

Figure S1 (a–c) QTLs related to gastro-intestinal nematode resistance in sheep (d) Chromosome-wise distribution of QTLs related to parasite resistance traits in sheep and number of SNP loci investigated in the present study (e) Quantitative trait loci (QTL) map of chromosome 3 related to parasite resistance traits in sheep (QTL Source data: Animal QTLdb, http://www.animalgenome.org/cgi-bin/QTLdb/index).
(TIF)

Figure S2 **Distribution of allele sharing distance (IBS) between pairs of individuals.** Distance was plotted separately where pairs were drawn from within the same breed (blue bars) and from across the breeds (red bars).
(TIF)

Figure S3 **Bayesian clustering of 713 sheep based on genotype data at (a) 18 non-neutral SNP loci (b) 23 neutral SNP loci under assumption of 2 to 6 clusters without a priori population information.** The breed names are given below the box plot and the geographical origin indicated above the box plot with the individuals of different breeds separated by vertical black lines.
(TIF)

Table S1 **Details of SNPs identified** ***in silico*** **within TLR genes of sheep.**
(DOCX)

Table S2 **Pairwise F_ST_ (lower triangle) and allele sharing distance (upper triangle) among different sheep breeds.** (BAN-Bangladeshi; BER-Bergschaf; COR-Corriedale; HAM-Hamdani; IFT-Indonesian Fat Tailed; ITT-Indonesian Thin Tailed; JUN-Junin; KAC-Kachchi; KAJ-Kajli; KAR-Karakachanska; KUL-Karakul; KSF-Krainer Steinschaf; MRS-Madras Red; MEC-Mecheri; MUF-Mouflon; NEL-Nellore; PAM-Pampinta; PAT-Pattanam; SHA-Shal; SHU-Shumenska; TEX-Texel; THA-Thalli).
(DOCX)

Table S3 **Global F-Statistics among different sheep populations at 41 SNP loci.**
(DOCX)

Table S4 **Results of Ewens Watterson Neutrality test at different SNP loci in various sheep breeds (1 – Significantly deviate from neutrality; 0 – No significant deviation from neutrality).**
(DOCX)

Table S5 **Analysis of molecular variance among different sheep breeds based on (right) genotypes at 18 non-neutral SNP loci and (left) genotypes at 23 neutral SNP loci.**
(DOCX)
