# Supplementary figures and images for: Candidate Gene Approach for Parasite Resistance in Sheep – Variation in Immune Pathway Genes and Association with Fecal Egg Count
Source: PLoS One. 2014 Feb 12;9(2):e88337. doi: 10.1371/journal.pone.0088337 (PMC3922807; doi:10.1371/journal.pone.0088337)

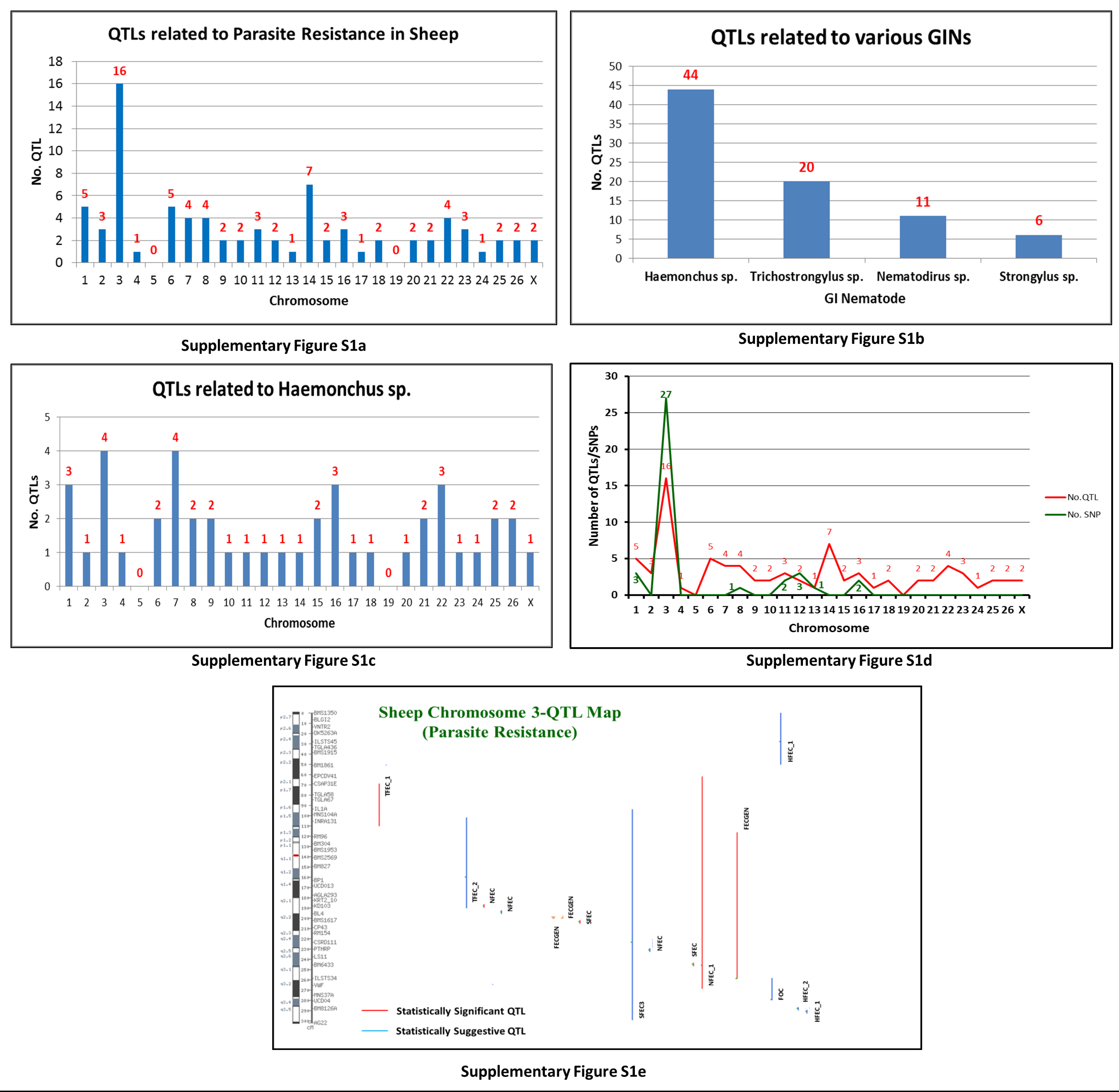

Supplement: Figure S1 — (a–c) QTLs related to gastro-intestinal nematode resistance in sheep (d) Chromosome-wise distribution of QTLs related to parasite resistance traits in sheep and number of SNP loci investigated in the present study (e) Quantitative trait loci (QTL) map of chromosome 3 related to parasite resistance traits in sheep (QTL Source data: Animal QTLdb, http://www.animalgenome.org/cgi-bin/QTLdb/index). (TIF) [file pone.0088337.s001.tif]

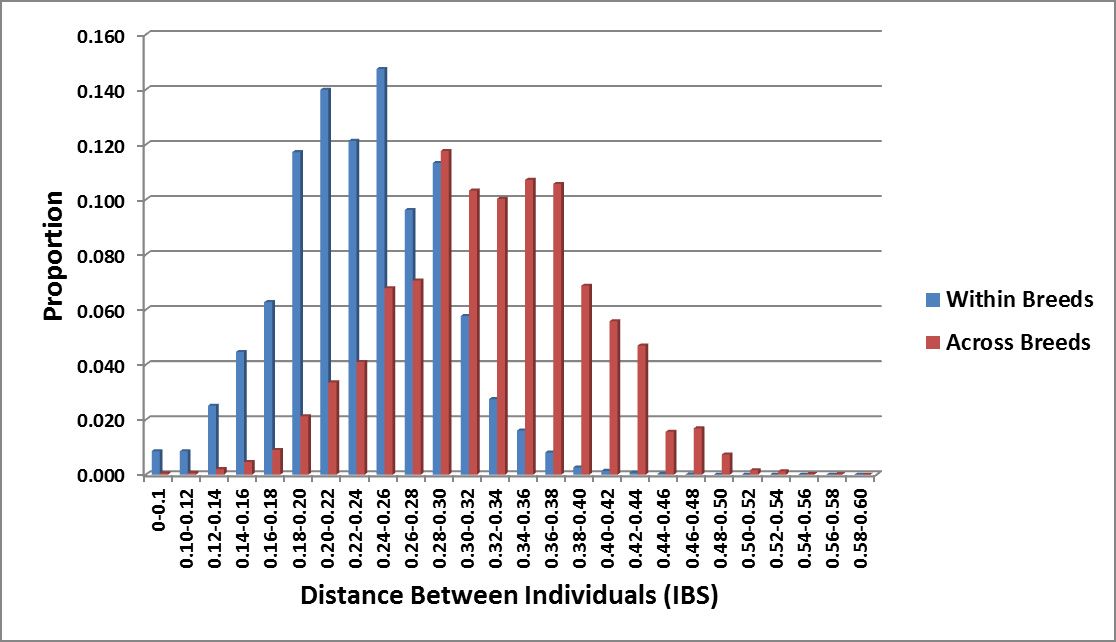

Supplement: Figure S2 — Distribution of allele sharing distance (IBS) between pairs of individuals. Distance was plotted separately where pairs were drawn from within the same breed (blue bars) and from across the breeds (red bars). (TIF) [file pone.0088337.s002.tif]

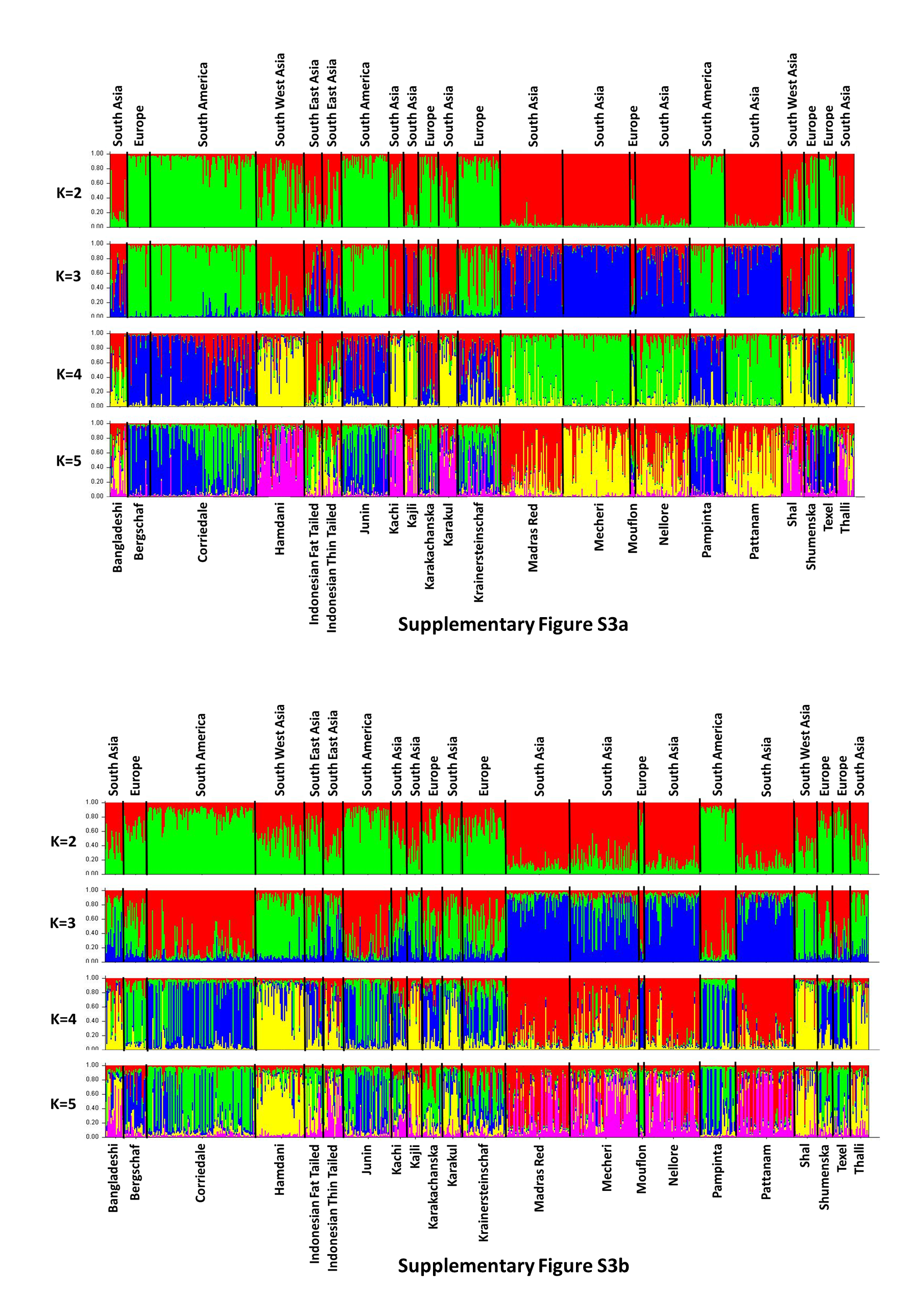

Supplement: Figure S3 — Bayesian clustering of 713 sheep based on genotype data at (a) 18 non-neutral SNP loci (b) 23 neutral SNP loci under assumption of 2 to 6 clusters without a priori population information. The breed names are given below the box plot and the geographical origin indicated above the box plot with the individuals of different breeds separated by vertical black lines. (TIF) [file pone.0088337.s003.tif]
