# Supplementary material for: Candidate Gene Approach for Parasite Resistance in Sheep – Variation in Immune Pathway Genes and Association with Fecal Egg Count
Source: PLoS One. 2014 Feb 12;9(2):e88337. doi: 10.1371/journal.pone.0088337 (PMC3922807; doi:10.1371/journal.pone.0088337)
Supplement: Table S1 — Details of SNPs identified in silico within TLR genes of sheep. (DOCX) [file pone.0088337.s004.docx]

Supplementary Table S1. Details of SNPs identified *in silico* within TLR genes of sheep

| Gene | Location | Name of SNP | Nulceotide change | Amino acid change | Position-Ref. Seq | Sequence_ID | Nucleotide |
| --- | --- | --- | --- | --- | --- | --- | --- |
| TLR1 |  | TLR1-T521C | T-C | S-P | AM 981299 | AM 981299 | T |
|  |  |  |  |  |  | NM001135060 | T |
|  |  |  |  |  |  | AM231298 | T |
|  |  |  |  |  |  | AY957612 | T |
|  |  |  |  |  |  | EF681961 | T |
|  |  |  |  |  |  | EF681962 | C |
| TLR1 |  | TLR1-A731G | A-G | T-A | AM 981299 | AM 981299 | A |
|  |  |  |  |  |  | NM001135060 | A |
|  |  |  |  |  |  | AM231298 | - |
|  |  |  |  |  |  | AY957612 | - |
|  |  |  |  |  |  | EF681961 | G |
|  |  |  |  |  |  | EF681962 | G |
| TLR2 |  | TLR2-A420G | A-G | E-G | AM981300 | AM981300 | A |
|  |  |  |  |  |  | DQ890157 | G |
|  |  |  |  |  |  | EU580543 | G |
|  |  |  |  |  |  | NM00104823 | G |
|  |  |  |  |  |  | AM117123 | - |
|  |  |  |  |  |  | EF681963 | - |
|  |  |  |  |  |  | EF681964 | - |
|  |  |  |  |  |  | EF681965 | - |
| TLR2 |  | TLR2-T2036C | T-C | F-L | AM981300 | AM981300 | T |
|  |  |  |  |  |  | DQ890157 | C |
|  |  |  |  |  |  | EU580543 | T |
|  |  |  |  |  |  | NM00104823 | C |
|  |  |  |  |  |  | AM117123 | T |
|  |  |  |  |  |  | EF681963 | T |
|  |  |  |  |  |  | EF681964 | T |
|  |  |  |  |  |  | EF681965 | T |
| TLR3 |  | TLR3-A342G | A-G | K-R | AM981301 | AM981301 | A |
|  |  |  |  |  |  | NM001135928 | A |
|  |  |  |  |  |  | GU936200 | A |
|  |  |  |  |  |  | GU936201 | G |
|  |  |  |  |  |  | GU936202 | A |
| TLR3 |  | TLR3-A635G | A-G | T-A | AM981301 | AM981301 | A |
|  |  |  |  |  |  | NM001135928 | A |
|  |  |  |  |  |  | GU936200 | G |
|  |  |  |  |  |  | GU936201 | A |
|  |  |  |  |  |  | GU936202 | A |
| TLR5 |  | TL45-G2093A | G-A | C-Y | NM001135926 | NM001135926 | G |
|  |  |  |  |  |  | AM981303 | G |
|  |  |  |  |  |  | AM981301 | A |
|  |  |  |  |  |  | AY957616 | - |
| TLR5 |  | TLR5-G2276C | G-C | S-T | NM001135926 | NM001135926 | G |
|  |  |  |  |  |  | AM981303 | G |
|  |  |  |  |  |  | AM981301 | G |
|  |  |  |  |  |  | AY957616 | C |
| TLR6 |  | TLR6-G1402A | G-A | R-Q | NM001135927 | NM001135927 | G |
|  |  |  |  |  |  | AM981304 | G |
|  |  |  |  |  |  | AM231302 | A |
| TLR7 |  | TLR7-A2543G | A-G | K-R | HQ529279 | HQ529279 | A |
|  |  |  |  |  |  | NM001135059 | A |
|  |  |  |  |  |  | GQ175932 | A |
|  |  |  |  |  |  | GQ175933 | G |
| TLR8 |  | TLR8-T1045C | T-C | Y-H | AM981306 | AM981306 | T |
|  |  |  |  |  |  | FJ905847 | T |
|  |  |  |  |  |  | GU936186 | T |
|  |  |  |  |  |  | GU936187 | T |
|  |  |  |  |  |  | NM001135929 | T |
|  |  |  |  |  |  | GQ175943 | T |
|  |  |  |  |  |  | GQ175944 | C |
| TLR8 |  | TLR8-T2504C | T-C | M-T | AM981306 | AM981306 | T |
|  |  |  |  |  |  | FJ905847 | T |
|  |  |  |  |  |  | GU936186 | C |
|  |  |  |  |  |  | GU936187 | T |
|  |  |  |  |  |  | NM001135929 | T |
|  |  |  |  |  |  | GQ175943 | - |
|  |  |  |  |  |  | GQ175944 | - |
| TLR9 |  | TLR9-A556G | A-G | R-A | ss184956560 | - | - |
| TLR9 |  | TLR9-C646T | C-T | R-C | ss184956561 | - | - |
