# Supplementary material for: Candidate Gene Approach for Parasite Resistance in Sheep – Variation in Immune Pathway Genes and Association with Fecal Egg Count
Source: PLoS One. 2014 Feb 12;9(2):e88337. doi: 10.1371/journal.pone.0088337 (PMC3922807; doi:10.1371/journal.pone.0088337)
Supplement: Table S2 — Pairwise FST (lower triangle) and allele sharing distance (upper triangle) among different sheep breeds. (BAN-Bangladeshi; BER-Bergschaf; COR-Corriedale; HAM-Hamdani; IFT-Indonesian Fat Tailed; ITT-Indonesian Thin Tailed; JUN-Junin; KAC-Kachchi; KAJ-Kajli; KAR-Karakachanska; KUL-Karakul; KSF-Krainer Steinschaf; MRS-Madras Red; MEC-Mecheri; MUF-Mouflon; NEL-Nellore; PAM-Pampinta; PAT-Pattanam; SHA-Shal; SHU-Shumenska; TEX-Texel; THA-Thalli). (DOCX) [file pone.0088337.s005.docx]

Supplementary Table S2. Pairwise F_ST_ (lower triangle) and allele sharing distance (upper triangle) among different sheep breeds (BAN-Bangladeshi; BER-Bergschaf; COR-Corriedale; HAM-Hamdani; IFT-Indonesian Fat Tailed; ITT-Indonesian Thin Tailed; JUN-Junin; KAC-Kachchi; KAJ-Kajli; KAR-Karakachanska; KUL-Karakul; KSF-Krainer Steinschaf; MRS-Madras Red; MEC-Mecheri; MUF-Mouflon; NEL-Nellore; PAM-Pampinta; PAT-Pattanam; SHA-Shal; SHU-Shumenska; TEX-Texel; THA-Thalli)

|  | BAN | BER | COR | HAM | IFT | ITT | JUN | KAC | KAJ | KAR | KUL | KSF | MRS | MEC | MUF | NEL | PAM | PAT | SHA | SHU | TEX | THA |
| --- | --- | --- | --- | --- | --- | --- | --- | --- | --- | --- | --- | --- | --- | --- | --- | --- | --- | --- | --- | --- | --- | --- |
| BAN | 0.000 | 0.282 | 0.236 | 0.158 | 0.171 | 0.145 | 0.211 | 0.223 | 0.157 | 0.200 | 0.149 | 0.242 | 0.181 | 0.153 | 0.336 | 0.155 | 0.258 | 0.168 | 0.168 | 0.216 | 0.252 | 0.152 |
| BER | 0.258 | 0.000 | 0.162 | 0.221 | 0.272 | 0.238 | 0.164 | 0.283 | 0.254 | 0.197 | 0.211 | 0.142 | 0.300 | 0.315 | 0.375 | 0.290 | 0.164 | 0.299 | 0.242 | 0.190 | 0.232 | 0.247 |
| COR | 0.210 | 0.109 | 0.000 | 0.213 | 0.204 | 0.207 | 0.074 | 0.265 | 0.257 | 0.135 | 0.207 | 0.137 | 0.307 | 0.311 | 0.328 | 0.291 | 0.138 | 0.313 | 0.264 | 0.155 | 0.166 | 0.244 |
| HAM | 0.115 | 0.194 | 0.181 | 0.000 | 0.206 | 0.186 | 0.200 | 0.213 | 0.167 | 0.174 | 0.134 | 0.185 | 0.227 | 0.222 | 0.330 | 0.212 | 0.208 | 0.234 | 0.154 | 0.178 | 0.218 | 0.144 |
| IFT | 0.114 | 0.268 | 0.166 | 0.168 | 0.000 | 0.110 | 0.204 | 0.276 | 0.221 | 0.202 | 0.208 | 0.193 | 0.224 | 0.189 | 0.287 | 0.184 | 0.227 | 0.209 | 0.225 | 0.191 | 0.235 | 0.180 |
| ITT | 0.068 | 0.223 | 0.181 | 0.130 | 0.042 | 0.000 | 0.200 | 0.248 | 0.173 | 0.205 | 0.163 | 0.209 | 0.173 | 0.159 | 0.341 | 0.143 | 0.237 | 0.168 | 0.187 | 0.191 | 0.241 | 0.162 |
| JUN | 0.192 | 0.110 | 0.019 | 0.168 | 0.162 | 0.162 | 0.000 | 0.266 | 0.244 | 0.128 | 0.181 | 0.163 | 0.308 | 0.292 | 0.341 | 0.271 | 0.122 | 0.295 | 0.240 | 0.153 | 0.176 | 0.240 |
| KAC | 0.213 | 0.297 | 0.253 | 0.203 | 0.276 | 0.227 | 0.259 | 0.000 | 0.213 | 0.276 | 0.214 | 0.261 | 0.248 | 0.263 | 0.376 | 0.261 | 0.265 | 0.280 | 0.251 | 0.231 | 0.225 | 0.239 |
| KAJ | 0.105 | 0.256 | 0.259 | 0.122 | 0.186 | 0.122 | 0.240 | 0.197 | 0.000 | 0.230 | 0.121 | 0.248 | 0.170 | 0.157 | 0.364 | 0.162 | 0.247 | 0.182 | 0.133 | 0.203 | 0.263 | 0.115 |
| KAR | 0.149 | 0.164 | 0.076 | 0.135 | 0.139 | 0.139 | 0.071 | 0.268 | 0.237 | 0.000 | 0.207 | 0.116 | 0.265 | 0.259 | 0.341 | 0.240 | 0.186 | 0.263 | 0.239 | 0.163 | 0.206 | 0.207 |
| KUL | 0.085 | 0.178 | 0.177 | 0.074 | 0.163 | 0.102 | 0.155 | 0.181 | 0.056 | 0.170 | 0.000 | 0.219 | 0.224 | 0.201 | 0.340 | 0.212 | 0.192 | 0.229 | 0.133 | 0.184 | 0.223 | 0.147 |
| KSF | 0.205 | 0.105 | 0.090 | 0.138 | 0.153 | 0.169 | 0.107 | 0.258 | 0.230 | 0.060 | 0.171 | 0.000 | 0.257 | 0.268 | 0.306 | 0.246 | 0.180 | 0.257 | 0.235 | 0.155 | 0.224 | 0.206 |
| MRS | 0.154 | 0.332 | 0.333 | 0.226 | 0.231 | 0.148 | 0.329 | 0.242 | 0.133 | 0.291 | 0.207 | 0.281 | 0.000 | 0.123 | 0.352 | 0.107 | 0.353 | 0.107 | 0.208 | 0.261 | 0.286 | 0.174 |
| MEC | 0.129 | 0.358 | 0.325 | 0.235 | 0.181 | 0.124 | 0.310 | 0.308 | 0.129 | 0.280 | 0.200 | 0.288 | 0.079 | 0.000 | 0.361 | 0.075 | 0.318 | 0.064 | 0.203 | 0.264 | 0.288 | 0.166 |
| MUF | 0.367 | 0.425 | 0.356 | 0.342 | 0.338 | 0.362 | 0.374 | 0.431 | 0.461 | 0.393 | 0.382 | 0.326 | 0.407 | 0.441 | 0.000 | 0.349 | 0.347 | 0.360 | 0.359 | 0.328 | 0.334 | 0.338 |
| NEL | 0.110 | 0.316 | 0.297 | 0.194 | 0.164 | 0.096 | 0.276 | 0.259 | 0.120 | 0.228 | 0.183 | 0.244 | 0.051 | 0.028 | 0.392 | 0.000 | 0.311 | 0.068 | 0.185 | 0.245 | 0.267 | 0.161 |
| PAM | 0.282 | 0.131 | 0.093 | 0.191 | 0.245 | 0.251 | 0.077 | 0.314 | 0.296 | 0.144 | 0.199 | 0.138 | 0.396 | 0.389 | 0.469 | 0.362 | 0.000 | 0.332 | 0.255 | 0.180 | 0.209 | 0.245 |
| PAT | 0.130 | 0.345 | 0.323 | 0.238 | 0.192 | 0.131 | 0.309 | 0.293 | 0.146 | 0.269 | 0.220 | 0.278 | 0.054 | 0.020 | 0.419 | 0.017 | 0.394 | 0.000 | 0.205 | 0.262 | 0.299 | 0.172 |
| SHA | 0.128 | 0.264 | 0.262 | 0.097 | 0.192 | 0.138 | 0.238 | 0.242 | 0.086 | 0.231 | 0.078 | 0.227 | 0.210 | 0.219 | 0.398 | 0.178 | 0.288 | 0.228 | 0.000 | 0.204 | 0.275 | 0.161 |
| SHU | 0.173 | 0.154 | 0.105 | 0.123 | 0.134 | 0.132 | 0.103 | 0.223 | 0.181 | 0.103 | 0.129 | 0.096 | 0.260 | 0.269 | 0.367 | 0.228 | 0.149 | 0.259 | 0.169 | 0.000 | 0.208 | 0.199 |
| TEX | 0.245 | 0.232 | 0.123 | 0.197 | 0.232 | 0.219 | 0.128 | 0.219 | 0.283 | 0.179 | 0.222 | 0.202 | 0.339 | 0.340 | 0.438 | 0.294 | 0.224 | 0.335 | 0.282 | 0.172 | 0.000 | 0.261 |
| THA | 0.071 | 0.224 | 0.221 | 0.086 | 0.145 | 0.097 | 0.211 | 0.196 | 0.049 | 0.193 | 0.073 | 0.185 | 0.137 | 0.139 | 0.388 | 0.129 | 0.252 | 0.139 | 0.122 | 0.164 | 0.271 | 0.000 |
