# Supplementary material for: Candidate Gene Approach for Parasite Resistance in Sheep – Variation in Immune Pathway Genes and Association with Fecal Egg Count
Source: PLoS One. 2014 Feb 12;9(2):e88337. doi: 10.1371/journal.pone.0088337 (PMC3922807; doi:10.1371/journal.pone.0088337)
Supplement: Table S3 — Global F-Statistics among different sheep populations at 41 SNP loci. (DOCX) [file pone.0088337.s006.docx]

Supplementary Table S3. Global F-Statistics among different sheep populations at 41 SNP loci

| Allele | FIT | FST | FIS | Allele | FIT | FST | FIS |
| --- | --- | --- | --- | --- | --- | --- | --- |
| ANKRD52_113 | 0.061 | 0.144 | -0.097 | PTPN6_398 | 0.145 | 0.145 | 0.001 |
| TARBP2_97 | 0.543 | 0.507 | 0.072 | PTPRB_141 | 0.148 | 0.129 | 0.022 |
| CLEC1A_134 | 0.139 | 0.086 | 0.058 | USP44_252 | 0.104 | 0.170 | -0.079 |
| CSF2RB_279 | 0.048 | 0.070 | -0.024 | SMCR7L_517 | 0.268 | 0.094 | 0.192 |
| CSF2RB_557 | 0.312 | 0.122 | 0.216 | ACVRL1_445 | 0.385 | 0.374 | 0.018 |
| ZDHHC17_190 | 0.124 | 0.083 | 0.045 | SLC11A2_174 | 0.109 | 0.066 | 0.046 |
| EM4b_574 | 0.114 | 0.042 | 0.076 | STAT3_138 | 0.014 | 0.065 | -0.054 |
| ESYT1_157 | 0.123 | 0.146 | -0.028 | TIMP3_716 | 0.159 | 0.156 | 0.003 |
| CSRNP2_65 | 0.214 | 0.232 | -0.024 | ZBTB39_51 | 0.060 | 0.083 | -0.026 |
| GLI1_253 | 0.361 | 0.394 | -0.053 | TLR5_2276 | 0.280 | 0.327 | -0.069 |
| GLI1_576 | 0.410 | 0.372 | 0.060 | TLR7_2491 | 0.516 | 0.303 | 0.305 |
| GPR84_520 | 0.239 | 0.286 | -0.066 | TLR8_1045 | 0.435 | 0.116 | 0.361 |
| IL2RA_388 | 0.119 | 0.173 | -0.066 | LEPR_260 | 0.178 | 0.101 | 0.086 |
| IL6R_227 | 0.302 | 0.302 | 0.000 | PIK3CD_443 | 0.371 | 0.364 | 0.012 |
| IL20RA_422 | 0.168 | 0.139 | 0.034 | IL10_82 | 0.239 | 0.251 | -0.017 |
| STAT2_486 | 0.145 | 0.224 | -0.102 | IL2RB_180 | 0.436 | 0.338 | 0.149 |
| ITGA5_111 | 0.226 | 0.224 | 0.003 | PRLR_341 | 0.072 | 0.121 | -0.056 |
| ITGB7_538 | 0.204 | 0.205 | -0.001 | PRLR_729 | 0.103 | 0.069 | 0.036 |
| DDIT3_527 | 0.470 | 0.414 | 0.095 | FGD6_519 | 0.135 | 0.161 | -0.031 |
| NAV3_591 | 0.088 | 0.119 | -0.036 | STAT5B_385 | 0.017 | 0.110 | -0.104 |
| PIK3R3_498 | 0.222 | 0.173 | 0.059 | OVERALL | 0.227 | 0.213 | 0.018 |
