# Supplementary material for: Candidate Gene Approach for Parasite Resistance in Sheep – Variation in Immune Pathway Genes and Association with Fecal Egg Count
Source: PLoS One. 2014 Feb 12;9(2):e88337. doi: 10.1371/journal.pone.0088337 (PMC3922807; doi:10.1371/journal.pone.0088337)
Supplement: Table S5 — Analysis of molecular variance among different sheep breeds based on (right) genotypes at 18 non-neutral SNP loci and (left) genotypes at 23 neutral SNP loci. (DOCX) [file pone.0088337.s008.docx]

Supplementary Table S5. Analysis of molecular variance among different sheep breeds based on (right) genotypes at 18 non-neutral SNP loci and (left) genotypes at 23 neutral SNP loci

| **Source of variation** | **Based on genotype data at 18 non-neutral SNP loci** | | | | | | **Based on genotype data from 23 neutral SNP loci** | | | | | | |
| --- | --- | --- | --- | --- | --- | --- | --- | --- | --- | --- | --- | --- | --- |
|  | **d.f.** | **Sum of squares** | **Variance components** | | **% of variation** | **P-**  **value** | **d.f.** | **Sum of squares** | | **Variance components** | | **% of variation** | **P-value** |
| ***No Groupings*** | | | | | | | | | | | | | |
| Among populations | 21 | 1251.23 | 0.90 | Va | 23.94 | 0.000 | 21 | | 954.28 | 0.67 | Va | 18.63 | 0.000 |
| Within populations | 1404 | 3999.26 | 2.85 | Vb | 76.06 | 0.000 | 1404 | | 4119.74 | 2.93 | Vb | 81.37 | 0.000 |
| ***Grouping-I (Asia, Europe, South America)**** | | | | | | | | | | | | | |
| Among groups | 2 | 657.25 | 0.69 | Va | 17.13 | 0.001 | 2 | | 424.21 | 0.41 | Va | 11.01 | 0.000 |
| Among populations within groups | 19 | 593.98 | 0.47 | Vb | 11.84 | 0.000 | 19 | | 530.07 | 0.42 | Vb | 11.08 | 0.000 |
| Within populations | 1404 | 3999.26 | 2.85 | Vc | 71.03 | 0.000 | 1404 | | 4119.74 | 2.93 | Vc | 77.92 | 0.000 |
| ***Grouping-II (South Asia, South East Asia, South West Asian, Europe, South America)***** | | | | | | | | | | | | | |
| Among groups | 4 | 813.80 | 0.68 | Va | 17.30 | 0.000 | 4 | | 568.53 | 0.45 | Va | 12.09 | 0.000 |
| Among populations within groups | 17 | 437.44 | 0.38 | Vb | 9.79 | 0.000 | 17 | | 385.74 | 0.33 | Vb | 8.90 | 0.000 |
| Within populations | 1404 | 3999.26 | 0.85 | Vc | 72.90 | 0.000 | 1404 | | 4119.74 | 2.93 | Vc | 79.01 | 0.000 |

* Grouping-I: Asia – Bangladeshi, Madras Red, Mecheri, Pattanam, Nellore, Indonesian Fat Tailed, Indonesian Thin Tailed, Shal, Hamdani, Thalli, Kachi, Karakul, Kajli; Europe – Krainersteinschaf, Texel, Bergschaf, Mouflon, Karakachanska, Shumenska; South America – Junin, Pampinta, Corriedale

**Grouping-II: South Asia - Bangladeshi, Madras Red, Mecheri, Pattanam, Nellore, Thalli, Kachi, Karakul, Kajli; South East Asia - Indonesian Fat Tailed, Indonesian Thin Tailed; South West Asia – Hamdani, Shal; Europe – Krainersteinschaf, Texel, Bergschaf, Mouflon, Karakachanska, Shumenska; South America – Junin, Pampinta, Corriedale
